# Triatomines: Trypanosomatids, Bacteria, and Viruses Potential Vectors?

**DOI:** 10.3389/fcimb.2018.00405

**Published:** 2018-11-16

**Authors:** Caroline Barreto Vieira, Yanna Reis Praça, Kaio Luís da Silva Bentes, Paula Beatriz Santiago, Sofia Marcelino Martins Silva, Gabriel dos Santos Silva, Flávia Nader Motta, Izabela Marques Dourado Bastos, Jaime Martins de Santana, Carla Nunes de Araújo

**Affiliations:** ^1^Programa de Pós-Graduação em Ciências Médicas, Faculdade de Medicina, Universidade de Brasília, Brasília, Brazil; ^2^Laboratório de Interação Patógeno-Hospedeiro, Instituto de Ciências Biológicas, Departamento de Biologia Celular, Universidade de Brasília, Brasília, Brazil; ^3^Faculdade de Ceilândia, Universidade de Brasília, Brasília, Brazil

**Keywords:** kissing bugs, vector competence, infectious diseases, Chagas disease, pathogens

## Abstract

Triatominae bugs are the vectors of Chagas disease, a major concern to public health especially in Latin America, where vector-borne Chagas disease has undergone resurgence due mainly to diminished triatomine control in many endemic municipalities. Although the majority of Triatominae species occurs in the Americas, species belonging to the genus *Linshcosteus* occur in India, and species belonging to the *Triatoma rubrofasciata* complex have been also identified in Africa, the Middle East, South-East Asia, and in the Western Pacific. Not all of Triatominae species have been found to be infected with *Trypanosoma cruzi*, but the possibility of establishing vector transmission to areas where Chagas disease was previously non-endemic has increased with global population mobility. Additionally, the worldwide distribution of triatomines is concerning, as they are able to enter in contact and harbor other pathogens, leading us to wonder if they would have competence and capacity to transmit them to humans during the bite or after successful blood feeding, spreading other infectious diseases. In this review, we searched the literature for infectious agents transmitted to humans by Triatominae. There are reports suggesting that triatomines may be competent vectors for pathogens such as *Serratia marcescens, Bartonella*, and *Mycobacterium leprae*, and that triatomine infection with other microrganisms may interfere with triatomine-*T. cruzi* interactions, altering their competence and possibly their capacity to transmit Chagas disease.

## Introduction

Vector-borne diseases (VBDs) are human illnesses caused by parasites, viruses, and bacteria that are usually transmitted by a bloodsucking arthropod. According to the World Health Organization (WHO) VBDs together account for around 17% of all infectious diseases in the world, which are mainly distributed in tropical and subtropical areas, affecting poorest populations. However, environmental changes and globalization have placed many more people living in other parts of the world at risk of contracting VBDs. The emergence/re-emergence of these diseases is burdening the health systems in many countries due to possible epidemic outbreaks and their impact in morbidity and mortality (WHO, 2017).

**Figure 1:**
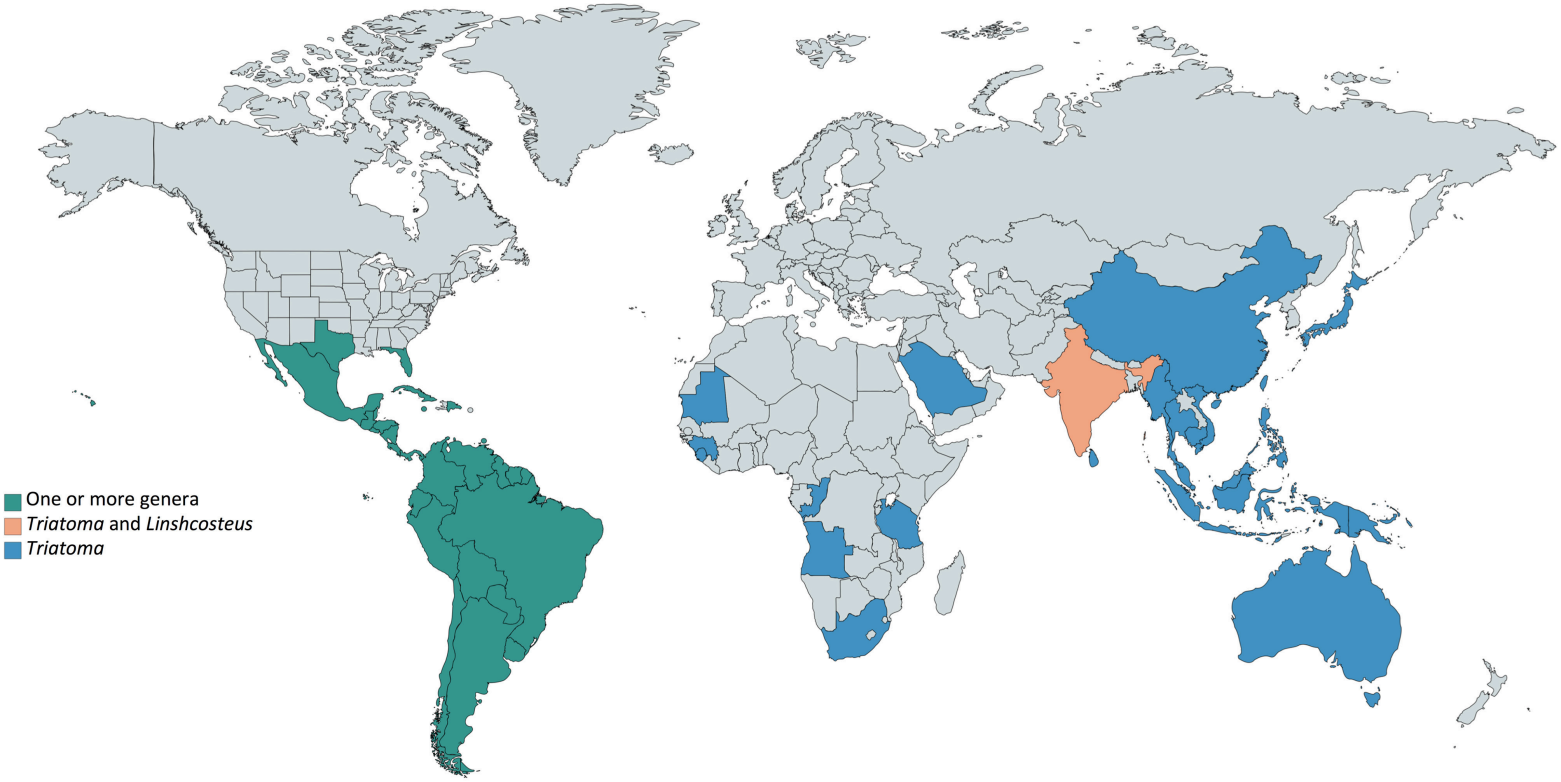
Worldwide Triatominae distribution. Triatominae species are present mainly in Americas (green), in which countries one or more genera can be found. Outside the American continent (blue), the only genus reported is *Triatoma*, except in India (soft orange), where *Triatoma* and *Linshcosteus* are reported.

Triatominae insects (Hemiptera: Reduviidae) (Lent and Wygodzinsky, 1979; Jurberg et al., 2014) are a diverse subfamily of ectoparasites mainly distributed across Americas (Guarneri et al., 2000; Otálora-Luna et al., 2015) (Figure 1). Six species belonging to the genus *Linshcosteus* occur in India, and species belonging to *Triatoma* have been also identified in Africa, the Middle East, South-East Asia and in the Western Pacific (Figure 1, Table S1). Currently, the 151 Triatominae species described are grouped in 17 genera and organized into 5 tribes: Aberproseniini, Bolboderini, Cavernicolini, Rhodiniini, and Triatomini (Table S1) (Alevi et al., 2014, 2016; Souza et al., 2016; da Rosa et al., 2017). They are typically found in a wide range of sylvatic environments frequently associated to several wild vertebrate hosts, possibly in their nests and burrows. Contact with humans occurs when men enter in forested areas or when they reach urban areas by flying and colonize human dwellings (Lazzari et al., 2013).

All species are considered able to transmit *Trypanosoma cruzi*, agent of Chagas disease. However those belonging to the genera *Triatoma, Rhodnius* and *Panstrongylus* have greatest epidemiological importance in Latin America (Cavassin et al., 2014), as they have adapted to colonize peridomestic and domestic environments, coming into close contact with domestic animals and humans. Domiciliation occurs through the loss of habitat due to irregular environment exploitation, such as deforestation and burnings (Dias and Schofield, 1998; Almeida et al., 2009) or through the loss of primary hosts, which can triggers a switch to accessible humans (Schofield, 1988).

*T. cruzi* and other pathogens infection within Triatominae colonies is due mainly to the vector blood feeding behavior. They are obligate hematophagous insects, exhibiting behavioral, morphological and molecular adaptations to feed on a variety of vertebrate blood throughout their life cycle (Lazzari et al., 2013), in which they undergo five nymphal stages before reaching adulthood (Jurberg et al., 2014). Therefore, their dietary behavior ensures the scenario to act as potential vectors of multiple pathogens to humans at any developmental stage. Moreover, exposure to triatomine bites and feces can result in immunological reactions (Walter et al., 2012), such as swelling, severe fever, itching at the bite site, and anaphylaxis (Klotz et al., 2010; Dujardin et al., 2015).

Thus, from the close relationship of Triatominae with vertebrate hosts, beyond *T. cruzi*, different triads of interaction (vector/pathogen/vertebrate host) may arise, allowing new infection routes for other microorganisms, such as bacteria and viruses, in cases Triatominae species show vector competence and vectorial capacity. Vectorial capacity is the ability of a vector to transmit a pathogen in a given locality at a definite time. It comprises the vector interactions with the infectious agent and with the vertebrate host(s), and is affected, for instance, by vector density, longevity, host preference, feeding behavior, and vector competence. Precisely, vector competence embodies vector-pathogen interactions, encompassing specifically the ability of a vector to become infected by and transmit a pathogen. It includes susceptibility to infection, permissiveness for pathogen reproduction and development, transmission efficiency and duration of extrinsic incubation period, which is the time from the uptake of an infectious meal to the time it is capable of transmitting the pathogen (Higgs and Beaty, 2004).

To be a competent vector, triatomine has to acquire the pathogenic microorganism from an infected host, to allow its replication in the midgut or to spread to the hemocoel in order to enter into the salivary glands. Hence, pathogens may be carried from the bug to the human host during insect bite by two different routes: via contaminated saliva released into host blood vessel or via contact with contaminated feces deposited at the host skin or near mucosa. It is worth mentioning that oral infection may also occur in case of food contamination with triatomine feces/saliva. An additional route of pathogen transmission may be xenodiagnosis, a diagnostic method that uses Triatominae nymphs as a biological culture medium for the detection of *T. cruzi* infection. Pathogens have been developing adaptations to exploit vector biology, behavior and ecology, and mainly, different mechanisms of transfer from one host to another to continue its species maintenance. Understanding all possible spreading routes of pathogens is crucial to prevent infectious diseases. Thus, considering the diversity of pathogens that Triatominae may acquire during blood feeding, the aim of this review was to examine published literature and summarize current information of triatomine competence to transmit, beyond *T. cruzi*, other pathogens to humans.

## Microorganisms transmitted through triatomine/man contact and their possible role in human health

### Trypanosomatids

#### *Trypanosoma cruzi* and chagas disease

The Trypanosomatidae family encompasses various obligate parasites, including the protozoa *T. cruzi* that causes Chagas disease (Hoare, 1972; Cavalier-Smith, 2016). The transmission of Chagas disease by Triatominae is very well reported in literature. Infection with vector-borne *T. cruzi* begins when metacyclic trypomastigotes, which are motile forms of the parasite, penetrate into the vertebrate host through the triatomine feces and urine. Once in the vertebrate host, these forms, which have evolved to survive inside host cells, infect nucleated cells. Within the cell, they differentiate into amastigotes in a phagosomal compartment known as the parasitophorous vacuole, escape to the cytoplasm and replicate asexually through longitudinal binary division to form several amastigotes. As the cell becomes full of amastigotes, these convert into trypomastigotes and breach it, invading adjacent tissues and spreading to distant sites through bloodstream and lymphatics. The parasite population expands due to repeated cycles of cell invasion and replication, which lead to immune responses and can give rise to Chagas-associated pathologies (Tyler and Engman, 2001).

**Figure 2:**
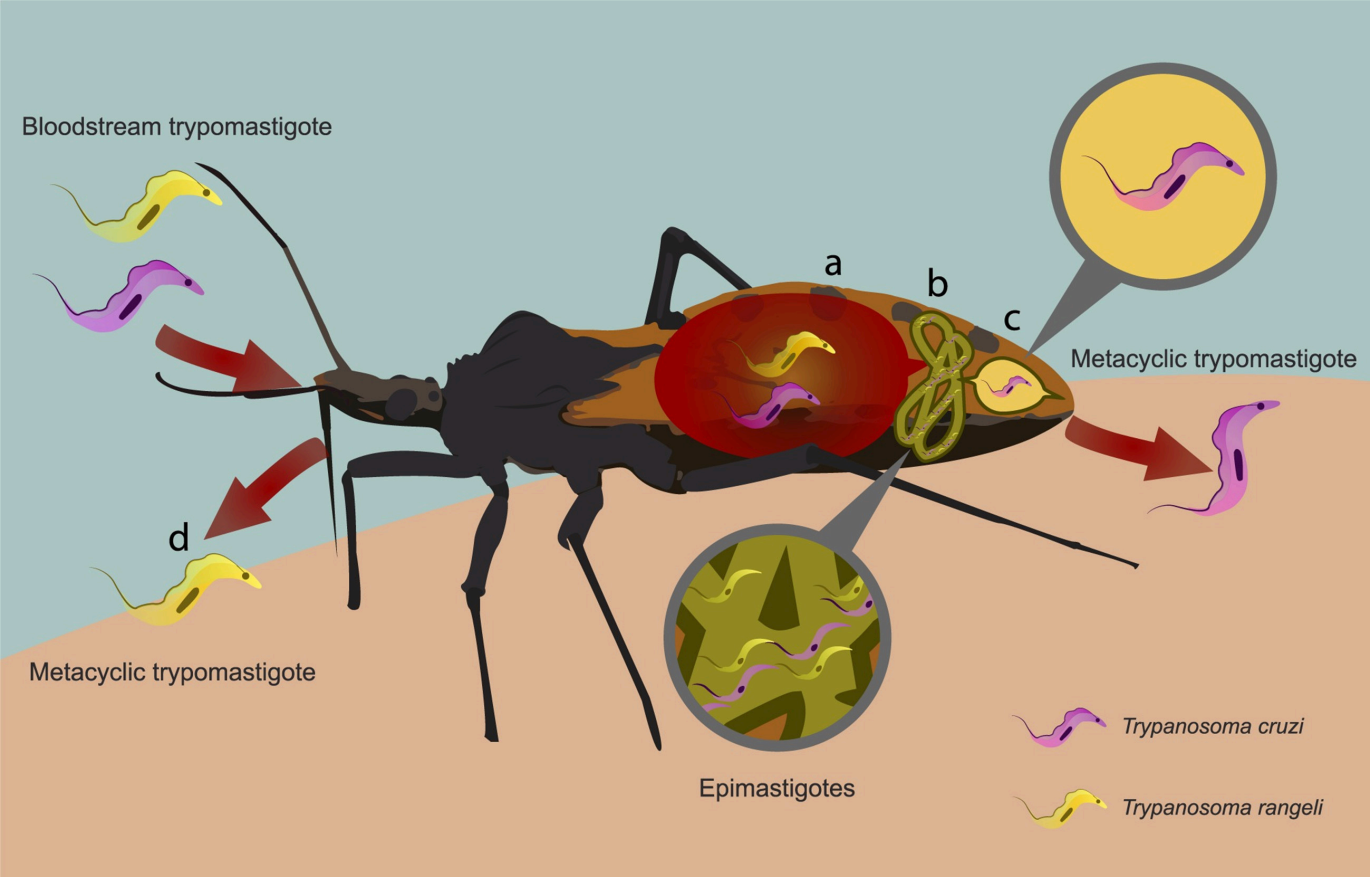
Schematic representation of *Trypanosoma cruzi* and *Trypanosoma rangeli* life cycles in the Triatominae. Triatomine infection occurs due to the ingestion of bloodstream trypomastigotes during the blood meal acquisition from a vertebrate host. **(A)** After ingestion, they transform into epimastigotes in the insect midgut. **(B)** *T. cruzi* epimastigotes multiply and then **(C)** migrate to the rectum where they differentiate into infective and non-replicative metacyclic trypomastigotes. These forms are eliminated with the triatomine feces and urine after a successful blood feeding. **(B)** *T. rangeli* epimastigotes reach the hemolymph and multiply, and then invade the salivary glands, differentiate into metacyclic trypomastigotes, **(D)** which are injected with saliva during the feeding process.

When triatomines take a blood meal, bloodstream trypomastigotes enter the midgut and differentiate into epimastigotes, amastigotes, and spheromastigotes (Rassi et al., 2010; Teixeira et al., 2011). Epimastigotes divide through longitudinal binary asexual reproduction repeatedly, which then migrate to the rectum, where they differentiate into infective and non-replicative metacyclic trypomastigotes, process called metacyclogenesis (Figure 2). *T. cruzi* infection intensity is triatomine species specific (Kollien and Schaub, 2000), as the conditions for its development in the gut vary substantially (Garcia et al., 2010). Therefore, the protozoan is capable of causing significant physiological changes such as delay in the development of *Triatoma infestans* nymphs (Schaub, 1989), decline in *Rhodnius prolixus* adult life span (Schaub, 1989) and of blood meal ingestion in infected *Triatoma dimidiata* nymphs (Schaub et al., 2011). Although epimastigotes and metacyclic trypomastigotes are eliminated along with the insect feces, only the latter are able to infect vertebrates (Schuster and Schaub, 2000), reach the vertebrate bloodstream through preexistent lesions or bite resultant breaches of the skin, or through mucosal tissues, repeating the life cycle (Billingsley and Downe, 1986; Figueiredo et al., 2000; Garcia et al., 2007). A balance among *T. cruzi* strain, inoculated burden, the innate and acquired immunological responses seems to be critical for the parasite control in the vertebrate host (Tarleton, 2007; Gil-Jaramillo et al., 2016).

Endemic to the continental part of Latin America, Chagas disease (American trypanosomiasis) affects approximately 8 million people worldwide (WHO, 2018a). It has been increasingly detected in non-endemic countries like USA, Canada, and countries in Europe and Western Pacific Region, due primarily to people movement between Latin America and the rest of the world, being an emerging infectious disease in these regions (WHO, 2018b). It is interesting to note that in the southern of USA recent surveillance data unveil Triatominae adults and nymphs in domestic and peridomestic areas, increasing human *T. cruzi* infection by vector-borne route, becoming Chagas disease a public health concern in this country (Montgomery et al., 2014; Edwards et al., 2017; Curtis-Robles et al., 2018).

In Vietnam, in the southeastern Asia, *Triatoma rubrofasciata* has been reported in high densities associated with rats and chickens close to human dwellings in rural and, specially, in urban areas. In the latter, *T. rubrofasciata* adults were reported hiding in people beds and feeding on them while sleeping, in buildings from the ground to the eighth floor. Although it is known this species transmit *T. cruzi* in Latin America and *Trypanosoma conorhini* worldwide, these trypanosomatids have not been detected in blood smears from individuals bitten by this bug. Closely related to *T. rubrofasciata* complex, *Linshcosteus* is uniquely distributed in Indian. Due to human migration and triatominae anthropophilia, *T. cruzi* infection emergence in Asia and India can arise (Dujardin et al., 2015).

Chagas disease warrants attention as it is considered the parasitic disease with the major socioeconomic impact in Latin America, being responsible for loss of productivity with an estimated value of US$ 1.2 billion annually (WHO/TDR, 2012).

#### Trypanosoma rangeli

Another parasite from the Trypanosomatidae family infecting humans and other vertebrates is *T. rangeli* (D'Alessandro, 1976; Guhl and Vallejo, 2003). Five phylogenetic lineages named TrA, TrB, TrC, TrD, and TrE have been identified to date, TrB being the most phylogenetically divergent one (Da Silva et al., 2004, 2009; Maia da Silva et al., 2004; Maia Da Silva et al., 2007; Ortiz et al., 2009; Caballero et al., 2015). TrA, TrB, and TrC lineages were reported to infect humans (Da Silva et al., 2004, 2009; Maia Da Silva et al., 2007; Ortiz et al., 2009; Pinto et al., 2012, 2015; Sincero et al., 2015; Dario et al., 2016, 2017). *T. rangeli* differs in life cycle, host infection and immune evasion both in triatomine and vertebrate hosts in comparison with *T. cruzi* (Espinosa-Álvarez et al., 2018).

Vector-borne *T. rangeli* transmission begins when metacyclic trypomastigotes penetrate into the vertebrate host through the triatomine saliva. Paim et al. (2013) showed that a 5th nymph of a *T. rangeli-*infected *R. prolixus* could carry up to 100,000 metacyclic trypomastigotes in its salivary glands. Approximately half of them can be released during blood feeding into the bite site (Ferreira Lde et al., 2015). Once in the vertebrate hosts, these forms may develop a multiplicative cycle. There is no strong evidence of reproductive forms of *T. rangeli* in these hosts, although some studies suggest their presence in vertebrates (de Scorza et al., 1986; Urdaneta-Morales and Tejero, 1986; Osorio et al., 1995; Ferreira Lde et al., 2015). Moreover, Ferreira *et al*. suggest that *T. rangeli* can hide in some tissues or organs, as it was rarely found circulating in mouse blood, even after an infected triatomine had injected a huge number of parasites (Ferreira Lde et al., 2015).

*T. rangeli* (sub-genera *Tejeraia*), as well as *Trypanosoma brucei* and *Trypansoma evansi* (sub-genera *Trypanozoon*), is classified as a Salivarian trypanosome because it multiplies in the anterior part of the vector digestive tract (Desquesnes et al., 2007). So, when triatomines feed on *T. rangeli-*infected hosts, trypomastigotes get into the midgut and differentiate into epimastigotes. These forms cross the gut epithelium, replicate in the hemolymph and then reach the salivary glands where they differentiate into metacyclic trypomastigotes. The cycle reinitiates when these forms are transmitted to mammals during the next triatomine bite (D'Alessandro, 1976) (Figure 2). *T. rangeli* is transmitted specially by Triatominae of the Rhodniini tribe. Its metacyclic trypomastigotes were already reported in *Rhodnius domesticus, Rhodnius nasutus, Rhodnius neglectus, Rhodnius pallescens, R. prolixus, Rhodnius robustus, Rhodnius brethesi, Rhodnius colombiensis, Rhodnius ecuadoriensis, Rhodnius dalessandroi, Rhodnius pictipes*, and *Rhodnius neivai* salivary glands (Guhl and Vallejo, 2003; Vallejo et al., 2009, 2015; Castro et al., 2017). Nevertheless, it was also reported in *Triatoma carrion, Panstrongylus chinai* and *Panstrongylus rufotuberculatus* species from the Triatominii tribe (Ocaña-Mayorga et al., 2015).

*T. rangeli* is capable of causing reduction in *R. prolixus* fecundity and fertility (Fellet et al., 2014), reduction in negative phototaxis and in *R. prolixus* expression of cGMP-dependent protein kinase gene, an ortholog of *Drosophila melanogaster foraging* gene reported to control the locomotion activity (Marliére et al., 2015), increase in mortality, defective, delayed or absent molts and tissue damages (Ferreira et al., 2010), among other effects in the infected triatomines (Guhl and Vallejo, 2003; Peterson and Graham, 2016). However, it seems *R. prolixus* co-infection with *T. rangeli* and *T. cruzi* ameliorate adverse effects of distinct infections, helping the insect to tolerate greater parasite infections (Peterson et al., 2016).

*T. rangeli* occurs from Central to South America (Grisard et al., 1999b). Although it is not considered a human pathogen, this trypanosomatid deserves attention as it shares biological characteristics, antigens, geographical distribution, insect and vertebrate hosts with *T. cruzi*. Mixed infections with *T. cruzi* are observed in humans, sylvatic and domestic mammals (Grisard et al., 1999b; Guhl and Vallejo, 2003). Thus, *T. rangeli* transmission by *T. cruzi* vectors is biologically and epidemiologically important due to crossed serological reactions (Guhl and Marinkelle, 1982; Guhl et al., 1987; Coura et al., 1996; Cuba Cuba, 1998; Calzada et al., 2010), what may result first in increased diagnostic test costs, and second in harms to human health due to Chagas disease drug toxicity where false positives are not detected (Grisard et al., 1999a). To circumvent this possibility, Moraes et al. evaluated the serological cross-reactivity between epimastigotes and trypomastigotes from *T. cruzi* and *T. rangeli* using sera of acute and chronic chagasic patients by indirect immunofluorescence and immunoblotting assays. These authors recommend retesting, in areas where there is overlapped distribution of both species, Chagas disease positive sera with *T. rangeli* trypomastigote antigens to avoid misdiagnosis (de Moraes et al., 2008).

### Bacteria

#### Serratia marcescens

*Serratia* genus (Enterobacteriaceae family) contains 14 species and 2 subspecies (Mahlen, 2011). They have been associated with human infections and reported as a symbiont in the microbiota of hematophagous insects (Grimont and Grimont, 1978; Grimont et al., 1979; Azambuja et al., 2004). In humans, *S. marcescens* is an important opportunistic gram-negative bacteria reported to cause wound, urinary tract, bloodstream and ocular infections, pneumonia and other respiratory diseases, meningitis, endocarditis, among other clinical diseases (Mahlen, 2011; González-Juarbe et al., 2015). The clinical and epidemiological significance are associated with sporadic hospital infection outbreaks due to *S. marcescens* strains ability to produce β-lactamases, which confer resistance to β-lactams and antiseptic agents available (Maragakis et al., 2008; Carvalho et al., 2010). Many outbreaks are frequently reported worldwide especially in neonatal intensive care units, where the bacteria usually spread rapidly and is associated with significant morbidity and mortality. The sources of the outbreaks have been associated to contaminated laryngoscope blades, hands, ventilator equipment, disinfectants, and breast pumps (Gransden et al., 1986; Jones et al., 2000; Sartor et al., 2000; Jang et al., 2001; Villari et al., 2001; Fleisch et al., 2002; Uduman et al., 2002; Cullen et al., 2005; Montagnani et al., 2015; Åttman et al., 2018).

Azambuja et al., isolated *S. marcescens* biotype A1a from the midgut of labroratory-reared *R. prolixus* (Azambuja et al., 2004), confirming previous reports on the occurrence of this species in triatomine gut (Figueiredo, 1995). Following feeding, this gram-negative bacillus rapidly multiplied and showed hemolytic and trypanolytic effects, the latter on *T. cruzi* Y strain but not on *T. cruzi* DM28c clone. Many strains of *S. marcescens* produce a reddish pigment named prodigiosin that is an important compound for the action against the parasite in the insect midgut (Azambuja et al., 2004). This report provides evidence that *R. prolixus* may be a competent vector for *S. marcescens*, as it was able to maintain infection in the gut, and that bacteria may be eliminated within the feces. Also suggests that *S. marcescens* may compete with *T. cruzi*, reducing *R. prolixus* competence for this trypanosomatid. On the other hand, *S. marcescens* biotype A1a did not raise triatomine mortality (Azambuja et al., 2004). The conduction of transmission studies from triatomines to mice would help to confirm the potential of *R. prolixus* to serve as vector for *S. marcescens*.

To date *S. marcescens* vector-borne transmission was not reported, but it is important to emphasize this possible mode of transmission. It is possible *S. marcescens* living in triatomine midgut to be acquired during the consumption of food containing the contaminated feces of an infected triatomine or maybe vector-borne through contaminated feces contact with open wounds or mucous membranes of a susceptible host. Future studies may evidence this possibility and indicate the consequences for human health.

#### Bartonella

*Bartonella* genus belongs to the Bartonellaceae family of the alphaproteobacteria (Regier et al., 2016), along with *Rickettsia* and *Brucella* (Sanchez Clemente et al., 2012) and contains 36 species, characterized by coccobacillary or bacillary rods. These pleomorphic fastidious gram-negative bacteria are considered a facultative intracellular pathogen (Maguiña and Gotuzzo, 2000). Human body lice, sandflies, cat fleas, flies and ticks are *Bartonella* vectors to humans and other mammals (Tsai et al., 2011). The vector-borne is the most relevant route of transmission (Pons et al., 2016).

A novel *Bartonella* genotype, named Candidatus *Bartonella rondoniensis*, closely related to *Bartonella bacilliformis* and *Bartonella ancashensis*, severe human pathogens, was described in the sylvatic triatomine *Eratyrus mucronatus* (Laroche et al., 2017). This new genotype is also related to *B. bovis* that has been observed to cause endocarditis in cats, which are accidental hosts of this species (Maillard et al., 2007). *E. mucronatus* feeds on bats and other small mammals (Castro et al., 2010), which are sources of virus and bacteria (Mühldorfer, 2013), including *Bartonella* (Laroche et al., 2017). Although sylvatic, this triatomine is attracted to artificial light sources (Castro et al., 2010) and has been reported in the proximity and inside houses. By now, Laroche et al. suggest that this species may host pathogenic bacteria (Laroche et al., 2017). An experimental model of infection would help to confirm if *E. mucronatus* is a competent vector for *Bartonella* species and act as their vector to humans. One possibility is that in these arthropods, *Bartonella* migrates to, replicate in the salivary glands and is transmitted during the bite.

In humans, *B. bacilliformis* is transmitted through the bite of infected *Lutzomyia verrucarum, Lutzomyia peruensis*, among other phlebotomine sandflies. This disease, named Carrion's disease, is endemic in South America and was the first human bartonellosis described. Carrion's disease can occur in two distinct concomitant or independent syndromes. The first is known as Oroya fever, which is characterized by hemolytic fever and bacteremia that without treatment can cause 40–88% death. Verruga peruana is the second syndrome, characterized by hemangiomas owed to endothelium infection (Minnick et al., 2014). *B. bacilliformis* closely related *B. ancashensis* was isolated from patients' blood with chronic verruga peruana, being suggested as a second agent of the disease (Mullins et al., 2015). Other *Bartonella* species may cause endocarditis, *B. quintana* causes trench fever and *B. henselae* causes cat scratch disease (Regier et al., 2016).

#### Mycobacterium leprae

*M. leprae* belongs to Mycobacteriaceae family and causes leprosy, a disfiguring chronic systemic infectious disease. Early diagnosis and the available treatment with multi-drug therapy significantly reduced the disease burden in recent decades. However, the lack of awareness about early signs of leprosy that contributes to a delay in diagnosis, and the persistent stigma and discrimination against affected people are factors that complicate the fight against leprosy. The global incidence of new cases was approximately 213,000 in 2014, and the highest prevalence rates are observed in Brazil, India and Indonesia, which together account for 81% of the newly diagnosed cases globally (WHO, 2016). To date, the exact mechanism of leprosy transmission is not completely understood, but it is supposed that transmission occurs due to the inhalation of infectious aerosols released by untreated cases of the disease or by direct contact from an infected person to a susceptible individual (Scollard et al., 2006; WHO, 2015). Interestingly, studies point out the viability of *M. leprae* outside human body and its existence in the environment, suggesting a different possibility in disease transmission (Desikan and Sreevatsa, 1995; Matsuoka et al., 1999; Turankar et al., 2012, 2016).

Recently, a report from Triatominae gut microbiomes revealed the presence of *Mycobacterium* in *Triatoma protracta* species microbiome profile (Rodríguez-Ruano et al., 2018). A further study evaluated the potential of *M. leprae* transmission by some insects and it was shown that *R. prolixus* might be able to transmit these bacteria, once they were present in *R. prolixus* feces (Neumann Ada et al., 2016). Perhaps the approximately 50% of Triatominae species found in Brazil, *Linshcosteus* (6 species) in India and *T. rubrofasciata* in Indonesia (Table S1) could help leprosy transmission in these three countries with the highest global incidences of the disease. McFadzean and Macdonald evaluated the possible role of mosquitoes and bed bugs in leprosy transmission by allowing infected and control insects to take a blood meal on volunteers and found no difference in transmission (McFadzean and Macdonald, 1961). Almost two decades later, the presence of *M. leprae* was reported in the proboscis, cuticle and blood smears from mosquitoes and ticks (de Souza-Araujo, 1942; Banerjee et al., 1991), suggesting arthropods could act as biological or mechanical vectors to these bacilli (Kirchheimer, 1976; Benchimol and Romero Sa, 2003).

Armadillos (*Dasypus novemcinctus*), which are parasitized by triatomines (Lainson et al., 1979), are a natural reservoir for *M. leprae* (Walsh et al., 1975; Smith et al., 1983) and also for *T. cruzi* (Lainson et al., 1979; Paige et al., 2002). Moreover, it is important to highlight that a study reported wild armadillos as well as patients with leprosy infected with the same strain of *M. leprae* (Truman et al., 2011). In this context, it is a topic of concern the overlapping geographic distribution of Triatominae species and the endemicity of leprosy in some regions, as triatomines that are transitioning from wild environments to the domiciliary ones may be a potential source of *M. leprae* transmission to humans (Neumann Ada et al., 2016), supporting that old hypothesis that leprosy can be vector-borne transmitted through the insect feces containing the bacteria when in contact with host wound or mucosa (Kirchheimer, 1976; Benchimol and Romero Sa, 2003; Neumann Ada et al., 2016).

### Virus

#### Human immunodeficiency virus (HIV) and hepatitis B virus (HBV)

HIV (genus *Lentivirus, Retroviridae* family) is own single-stranded RNA virus that infects human CD4^+^ T lymphocytes and macrophages. HIV genome contains nine ORFs that encode for 15 proteins (Frankel and Young, 1998), fundamental to replication and evasion from host defense. HIV causes infection in approximately 36.7 million people worldwide and was responsible for 1.0 million HIV-related causes deaths in 2016 (WHO, 2018c).

HBV (*Hepadnaviridae* family) infection causes acute and chronic hepatitis, and in severe forms, can lead to the development of cirrhosis and hepatocellular carcinoma (Liang, 2009). HBV causes infection in approximately 257 million people and was responsible for 887,000 deaths in 2015 (WHO, 2018d).

HIV- or HBV-infected patients were submitted to xenodiagnosis to investigate HIV or HBV possible transmission by triatomines (Granato et al., 1987; Nuzzo et al., 1998). For triatomines fed on AIDS patients in whose blood p24 HIV antigen was detected, no antigen was detected in the feces samples analyzed 24 h or 48 h post-blood meal (Nuzzo et al., 1998). For HBV, samples of feces and hemolymph were analyzed periodically and revealed that the hepatitis B surface antigen (HBsAg) was eliminated until 15 days post-blood meal. These authors concluded that although the HBV transit by the triatomine gastrointestinal tract, these insects may not yield efficient HBV infection (Granato et al., 1987).

In a previous study, human blood samples obtained from wild caught *Panstrongylus, Rhodnius*, and *Triatoma* were evaluated for the presence of HBsAg by haemagglutination and radioimmunoassay techniques. By the former, seven samples were presumably positive for HBsAg, but by the latter, only one, obtained from a *Triatoma sordida* specimen, was positive for the antigen (Candeias et al., 1976). These studies did not reveal a trend in viruses' transmission by triatomines, but instead highlight that efforts should be made to ensure greater consistence among studies that evaluate this task.

#### Triatoma virus

Non-enveloped *Triatoma virus* (TrV) was first identified in *T. infestans* species in Argentina (Muscio et al., 1987). TrV belongs to the genus *Triatovirus* (Agirre et al., 2011) of the *Dicistroviridae* family (Czibener et al., 2000), in which viruses own positive-sense single-stranded RNA. Its genome contains two open reading frames, separated by an intergenic region, coding structural, and non-structural polyproteins (Johnson and Christian, 1998; Sasaki et al., 1998; Czibener et al., 2000; Domier et al., 2000; Wilson et al., 2000).

So far, TrV is the only pathogenic virus described in Triatominae. TrV transmission among triatomines occurs vertically, through cannibalism or fecal-oral route (Muscio et al., 1987, 1988, 2000). It replicates in the intestinal epithelium cells of triatomines, causing leg paralyzes, delayed development, reduced fertility and death (Muscio et al., 1987, 1988; Rozas-Dennis and Cazzaniga, 2000).

TrV average prevalence in *T. infestans* is greater than of *T. cruzi* (Marti et al., 2009) and other microorganisms associated with triatomines (Muscio et al., 1997; Marti et al., 2009). Consequently, it has been considered that people living in Chagas disease endemic areas may have entered in contact with TrV through virus particles present in triatomine feces (Muscio et al., 2000). This supposition has been confirmed by the positive serology for anti-TrV antibodies in mice (Querido et al., 2013), hens (Muscio et al., 2000), and rabbits (Muscio et al., 1997) used to feed triatomines infected with TrV. Humans who live in endemic areas also showed positive TrV serology, among them 12.2% corresponded to healthy individuals (Querido et al., 2015), thus supporting the observation that TrV infection levels are higher/greater than *T. cruzi* infection levels in triatomines.

So far, the cases of TrV infection in vertebrates indicate that this virus is unable to replicate inside their cells (Muscio et al., 2000; Querido et al., 2013, 2015), and so may have no impact on human health. However, TrV may increase, indirectly, *T. cruzi* transmission to vertebrate hosts, strengthening triatomine vector competence for this trypanosomatid, although more studies are needed to confirm this hypothesis (Marti et al., 2017).

## Final considerations

In recent years, triatomines have undergone a major resurgence in the number of infestations, leading to re-emergence of *T. cruzi* vector transmission. Although the majority of Triatominae species occurs in the Americas, *Linshcosteus* spp. occur in India and *T. rubrofasciata* complex occurs in Africa, the Middle East, South-East Asia, and the Western Pacific countries (Dujardin et al., 2015). Although not all of Triatominae species are naturally infected with *T. cruzi*, there is the possibility of establishing vector transmission to areas where Chagas disease was previously non-endemic due to global population mobility. Furthermore, the resurgence and worldwide distribution of triatomines is concerning, considering they can enter in contact with other pathogens.

**Figure 3:**
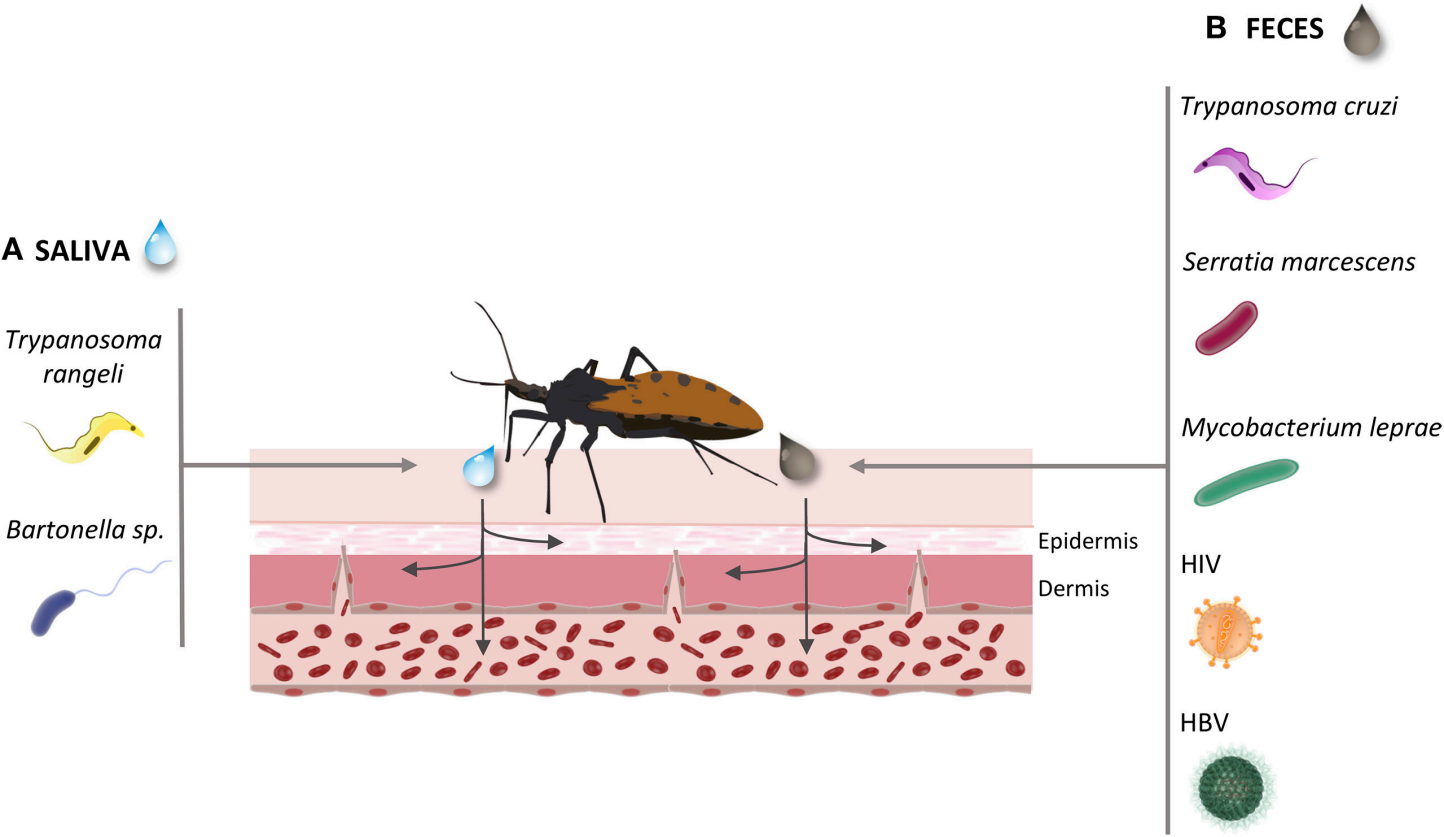
Common infection routes of the pathogens transmitted by Triatominae. During the Triatominae bite, the infected vector may transmit different pathogens to the host's vascular endothelium. **(A)** Via saliva inoculation: *Trypanosoma rangeli*; *Bartonella* sp. **(B)** Via feces penetration in mucous or open wound: *Trypanosoma cruzi*; *Serratia marcescens*; *Mycobacterium leprae*; Human immunodeficiency virus; Hepatitis B virus.

The possibility of these insects acting as vectors of other human pathogens aroused our curiosity. Triatomines are uncommon disease vectors since, except for salivarian trypanosomes as *T. rangeli*, pathogens seem unable to reach triatomine salivary glands in order to be transmitted by bite. Thus, the common route of transmission, as observed for *T. cruzi*, a stercorarian trypanosome, may be via contaminated feces released on skin wound or mucosa (Figure 3). We present reports of triatomine infected with vector pathogens (*T. rangeli* and TrV) already known to be transmitted to humans by triatomines, and of triatomines infected with human pathogens besides *T. cruzi* (the bacteria *S. marcescens, Bartonella* and *M. leprae;* and HIV and HBV) (Figure 3). Although no human viral disease can be attributed to Triatominae, TrV infection in triatomines may interfere with vector competence, augmenting the ability of triatomines to transmit Chagas disease.

Many factors may influence triatomine vector competence for transmitting pathogens to humans. The different regions in the gut themselves can bear unfavorable conditions, for instance pH, oxygen content, digestive enzymes, immune-related molecules, peristalsis, symbionts, and transient microorganisms (Douglas, 2015). Being competent does not mean to have the capacity to disseminate diseases, as vector competence is only one factor influencing vectorial capacity (Higgs and Beaty, 2004). Thus, further investigation and establishment of adapted models need to be developed in order to broaden the knowledge of triatominae ability to act as vector of other human infectious diseases.

## Author contributions

CdA, CV, YP, and KB were involved in the conception of and wrote the manuscript. PS, SS, GS, FM, IB and JdS wrote the manuscript. PS, YP, and KB prepared the figures and tables. All the authors read and approved the final manuscript.

### Conflict of interest statement

The authors declare that the research was conducted in the absence of any commercial or financial relationships that could be construed as a potential conflict of interest.
