# Supplementary material for: Triatomines: Trypanosomatids, Bacteria, and Viruses Potential Vectors?
Source: Front Cell Infect Microbiol. 2018 Nov 16;8:405. doi: 10.3389/fcimb.2018.00405 (PMC6250844; doi:10.3389/fcimb.2018.00405)
Supplement: Supplementary file 1 [file Table_1.docx]

Supplementary Material

Article Title

Triatomines: trypanosomatids, bacteria and viruses potential vectors? A review

Caroline Barreto Vieira, Yanna Reis Praça, Kaio Luís da Silva Bentes, Paula Beatriz Santiago, Sofia Marcelino Martins Silva, Gabriel dos Santos Silva, Flávia Nader Motta, Izabela Marques Dourado Bastos, Jaime Martins de Santana, Carla Nunes de Araújo^*^

# * Correspondence: Carla Nunes de Araújo: [cnunes@unb.br](mailto:cnunes@unb.br)

**S1 Table. Triatomine species currently reported.**

| **Tribe** | **Genus** | **N. of species** | **Species** | **Medical importance** | **Geographical distribution** | **Cycle** | **Habitat / observation** | **Reference** |
| --- | --- | --- | --- | --- | --- | --- | --- | --- |
| Aberproseniini | *Alberprosenia* | 2 | - *A. goyovargasi* | No | VEN | S | Dead trees / Feed: reptiles | (Martínez and Carcavallo 1977) |
|  |  |  | *A. malheiroi* | No | BRA – PA | S | Palms and woodpecker nests | (Serra, Atzingen, and Serra 1987) |
| Bolboderini | *Bolbodera* | 1 | *B. scabrosa* | No | CUB | S | Arboreal rodents holes. | (Valdés 1910) |
|  | *Microtriatoma* | 2 | *M. borbai* | No | BRA - GO, MT,PR, RJ | S | Bromeliads, rodent holes and marsupials | (Lent and Wygodzinsky 1979) |
|  |  |  | *M. trinidadensis* | No | BOL, COL, GUF, PER, TTO, VEN and BRA - MT, PA, TO | S | Palms and hollow of trees. | (Lent and Wygodzinsky 1979) |
|  | *Parabelminus* | 2 | *P. carioca* | No | BRA – RJ | S | Palms. | (Lent 1943) |
|  |  |  | *P. yurupucu* | No | BRA- BA | S | Bromeliads, hollow of trees and rodent holes | (Lent and Wygodzinsky 1979) |
|  | *Belminus* | 8 | *B. corredori* | No | COL | S | Domiciliary | (Galvao and Angulo 2006) |
|  |  |  | *B. costaricensis* | No | CRI | U | U | (Herrer, Lent, and Wygodzinsky 1954) |
|  |  |  | *B. ferroae* | No | COL | D | Domiciliary / Feed: cockroaches | (Sandoval et al. 2007) |
|  |  |  | *B. herreri* | No | COL and PAN | S | Trees | (Lent and Wygodzinsky 1979) |
|  |  |  | *B. laportei* | No | BRA - PA | U | U | (Lent, Jurberg, and Carcavallo 1995) |
|  |  |  | *B. peruvianos* | No | PER | P | Peridomiciliary | (Herrer, Lent, and Wygodzinsky 1954) |
|  |  |  | *B. pittieri* | No | VEN | U | U | (Osuna and Ayala 1993) |
|  |  |  | *B. rugulosus* | No | COL and VEN | U | U | (Stål 1859) |
| Cavernicolini | *Cavernicola* | 2 | *C. lenti* | No | BRA - AM | S | Hollow of trees along with rodents and bats | (Barrett and Arias 1985) |
|  |  |  | *C. pilosa* | No | COL, ECU, GUF, PAN, PER, VEN and  BRA - BA, ES, GO, MT, MS, MG, PA, PR TO | S | Hollow of trees and caves inhabited by bats | (Barber 1937) |
| Rhodiniini | *Psammolestes* | 3 | *P. arthuri* | No | COL and VEN | S | Nests of birds (Passeriformes: Furnariidae) | (Pinto 1926) |
|  |  |  | *P. coreodes* | No | ARG, PRY, BOL and BRA - MS, MT | S | Nests of birds (Passeriformes: Furnariidae) | (Lent and Wygodzinsky 1979) |
|  |  |  | *P. tertius* | No | BRA - BA, CE, GO, DF, SP, MA, MG, MT, PA, BA, PE, PI, TO | S | Nests of birds (Passeriformes: Furnariidae) | (Lent and Jurberg 1965) |
|  | *Rhodnius* | 21 | *R. prolixus* | Yes | COL, ECU, GUY, GUF, SUR, TTO and VEN | D | Main vector of Central America and north of South America | (Stål 1859) |
|  |  |  | *R. amazonicus* | No | GUF and BRA – AM | U | U | (Almeida, Santos, and Sposina 1973) |
|  |  |  | *R. barretti* | Yes | COL, ECU and PER | P | Palms / High infection rate; similar morphology: *R. montenegrensis*, *marabaensis* and *robustus* | (Abad-Franch et al. 2013) |
|  |  |  | *R. brethesi* | Yes | VEN and BRA - AM, PA | P | Palms: *Leopoldina piassaba* | (Matta 1919) |
|  |  |  | *R. colombiensis* | Yes | COL | P/ OD | Palms and occasionally found in houses | (Mejia, Galvão, and Jurberg 1999) |
|  |  |  | *R. dalessandroi* | No | COL | S | Rarely found; palms /Similar morphology : *R. prolixus* | (Carcavallo and Barreto 1976) |
|  |  |  | *R. domesticus* | No | BRA - BA, ES, MG, RJ, PR, SC, SP | S | Palms and bromeliad | (Neiva and Pinto 1923b) |
|  |  |  | *R. ecuadoriensis* | Yes | ECU and PER | P / D | Palms, trees and houses. / High infection rate; Important vector in the region PER e ECU | (Lent and León 1958) |
|  |  |  | *R. marabaensis* | No | BRA- PA | S | Rarely found; palms | (Souza et al. 2016) |
|  |  |  | *R. milesi* | No | BRA – PA | S | Rarely found; palms | (Valente et al. 2001) |
|  |  |  | *R. montenegrensis* | No | BRA – RO | S | Rarely found; palms | (Rosa et al. 2012) |
|  |  |  | *R. nasutus* | Yes | BRA - BA, CE, MA, PB, PE, PI, RN | P / OD | Palms, occasionally found in hen house and houses | (Stål 1859) |
|  |  |  | *R. neglectus* | Yes | BRA - BA, DF, GO, MA, MT, MS, MG, PB, PE, PI, PR, SP, TO | P / D | Palms, occasionally in henhouse and houses. | (Lent 1954) |
|  |  |  | *R. neivai* | Yes | COL and VEN | P | Palms and occasionally in houses. | (Lent 1953b) |
|  |  |  | *R. pallencens* | Yes | BLZ, COL, CRI, NIC and PAN | P / D | Palms. / High infection rate; Important vector in Central America. | (Barber 1932) |
|  |  |  | *R. pictipes* | Yes | BLZ, COL, ECU, GUY, GUF, PER, SUR, TTO, VEN and BRA - AP, AM, MT, PA, PI, RO, TO | D | Palms and houses (adults). | (Lent and Wygodzinsky 1979) |
|  |  |  | *R. robustus* | Yes | BOL, COL, ECU, GUF, PER, VEN and BRA - AC, AM, MA, MT, PA, RO, TO | P/ OD | Palms and occasionally in houses / Similar morphology : *R.marabaensis* and *barrette* | (Larrousse 1927) |
|  |  |  | *R. stali* | Yes | BOL and BRA - MS and MT | P / OD | Palms / Process of domiciliation in Bolivia. | (Lent, Jurberg, and Galvão 1993) |
|  |  |  | *R. taquarussuensis* | Yes | BRA – MS | D | Houses / Similar morphology : *R. neglectus* and *milesi* | (da Rosa et al. 2017) |
|  |  |  | *R. zeledoni* | No | BRA – SE | U | Description based on only copy found. | (Jurberg, Rocha, and Galvão 2009) |
|  |  |  | *R. paraensis* | No | GUF and BRA – AM, PA | S | Rarely found, nests of rodents. | (Sherlock, Guitton, and Miles 1977) |
| Triatomini | *Dipetalogaster* | 1 | *D. maxima* | No | MEX | S / OD | Desert, between rocks and occasionally in houses. | (Uhler 1894) |
|  | *Eratyrus* | 2 | *E. cuspidatus* | Yes | COL, ECU, GUA, MEX, PAN, PER and VEN | P / OD | Trees inhabited by bats, palms and occasionally in houses. | (Stål 1859) |
|  |  |  | *E. mucronatus* | Yes | BOL, COL, ECU, GUA, GU, GUF, PAN and BRA - AM, MA, MT, PA, RO, TO | S | Caves, trees, palms, nests of mammals and occasionally in houses | (Stål 1859) |
|  | *Hermanlentia* | 1 | *H. matsunoi* | Yes | PER | S | Caves and holes | (Fernández-Loayza 1989) |
|  | *Linshcosteus* | 6 | *L. confumus* | No | IND | S | Rocks | (Ghauri 1976) |
|  |  |  | *L. carnifex* | No | IND | S | Rocks | (Distant 1903) |
|  |  |  | *L. chota* | No | IND | S | Rocks | (Lent and Wygodzinsky 1979) |
|  |  |  | *L. costalis* | No | IND | S | Rocks | (Ghauri 1976) |
|  |  |  | *L. kali* | No | IND | S | Rocks | (Lent and Wygodzinsky 1979) |
|  |  |  | *L. karupus* | No | IND | S | Rocks | (Galvão et al. 2002) |
|  | *Mepraia* | 3 | *M. gajardoi* | Yes | CHL | P / OD | Rocks and occasionally found in houses | (Frias, Henry, and Gonzalez 1998) |
|  |  |  | *M. parapatrica* | Yes | CHL | P/ OD | Rocks and occasionally found in houses | (Frías-Lasserre 2010) |
|  |  |  | *M. spinolai* | Yes | CHL | P / OD | Rocks and occasionally found in houses | (Porter 1934) |
|  | *Nesotriatoma* | 3 | *N. bruneri* | Yes | CUB | D | Houses / Similar Morfology : *Nasotriatoma flavida* | (Usinger 1944) |
|  |  |  | *N. flavida* | Yes | CUB | D | Houses | (Neiva 1911b) |
|  |  |  | *N. obscura* | Yes | JAM | U | U | (Maldonado and Farr 1962) |
|  | *Panstrongylus* | 14 | *P. megistus* | Yes | ARG, BOL, PRY, URY and BRA - BA, CE, DF, ES, GO, MA, MT, MS, PA, PB, PE, PR, PI, RJ, RO, RR, RS, SC, SE, SP, TO | D | Armadillo's hole, marsupials and henhouse. | (Lent and Wygodzinsky 1979) |
|  |  |  | *P. chinai* | Yes | ECU, PER and VEN | D | Peridomiciliary - henhouse and Wild. | (Lent and Wygodzinsky 1979) |
|  |  |  | *P. diasi* | Yes | BOL and BRA - BA, DF, ES, GO, MA, MT, MG, SP, TO | D | Wild | (Pinto and Lent 1946) |
|  |  |  | *P. geniculatus* | Yes | ARG, BOL, COL, CRI, ECU, GUF, GTM, GUY, MEX, NIC, PAN, PER,URY, VEN, TTO, and BRA, AC, AP, AM, BA, CE, PE, DF, ES, GO, MA, MT, MS, PA, PR, PI, RJ, RO, RR, SP, TO | D | Armadillo's hole | (Latreille 1811) |
|  |  |  | *P. guentheri* | Yes | ARG, BOL, PAN, URY and BRA - MS | P/ OD | Rarely found in nests of rodents and birds; Occasionally found in houses | (Berg 1879) |
|  |  |  | *P. howardi* | No | ECU | P / OD | Occasionally found in houses. Wild | (Lent and Wygodzinsky 1979) |
|  |  |  | *P. humeralis* | No | COL and PAN | D | Occasionally found in houses. Wild | (Usinger 1939) |
|  |  |  | *P. lenti* | Yes | BRA - BA and GO | D | Rarely found. Ocasionally found in houses. Wild | (Galvão and Palma 1968) |
|  |  |  | *P. lignarius* | No | ECU, PER, GUY, GUF, SUR, VEN and BRA - AM, MA, MT, PA, TO | P / OD | Palm, henhouse and Occasionally found in houses | (Lent and Wygodzinsky 1979) |
|  |  |  | *P. lutzi* | Yes | BRA - AL, BA, CE, MG, PB, PE,PI, RN, SE | D | Armadillos's hole and rocks | (Neiva and Pinto 1923b) |
|  |  |  | *P. martinezorum* | Yes | VEN | D | Wild | (Ayala 2009) |
|  |  |  | *P. mitarakaensis* | Yes | GUF | R | U | (Bérenger and Blanchet 2007) |
|  |  |  | *P. rufotuberculatus* | Yes | ARG, BOL, COL, CRI, ECU, GUF, MEX, PAN, PRY, PER, VEN and BRA - AM, PA, MT | S / OD | Palms, Armadillo's hole, bats and occasionally found in houses | (Lent and Wygodzinsky 1979) |
|  |  |  | *P. tupynambai* | Yes | URY and BRA – RS | P / OD | Rocks, rodents nests and occasionally found in houses | (Lent 1942) |
|  | *Paratriatoma* | 1 | *P. hirsuta* | No | EUA and MEX | S | Nests of arboreal rodents | (Barber 1938) |
|  | *Triatoma* | 74 | *T. amicitiae* | Yes | LKA | U | U | (Lent 1951b) |
|  |  |  | *T. arthurneivai* | No | BRA- MG | S | Rocks, rodents and lizard nests | (Lent and Martins 1940) |
|  |  |  | *T. bahiensis* | Yes | BRA – BA | D | Domiciliary Environment | (Sherlock and Serafim 1967) |
|  |  |  | *T. baratai* | Yes | BRA – MS | P | Caves e henhouse | (Carcavallo and Jurberg 2000) |
|  |  |  | *T. barberi* | Yes | MEX | D | Domiciliary Environment | (Usinger 1939) |
|  |  |  | *T. bassolsae* | Yes | MEX | D | Domiciliary Environment | (Aguilar et al. 1999) |
|  |  |  | *T. bolivari* | Yes | MEX | U | U | (Carcavallo et al. 1967) |
|  |  |  | *T. boliviana* | No | BOL | D | Domiciliary Environment | (Martinez et al. 2007) |
|  |  |  | *T. bouvieri* | Yes | PHL, VNM and Nicobar Islands | U | U | (Larrousse 1924) |
|  |  |  | *T. brailovskyi* | Yes | MEX | U | U | (Martínez, Carcavallo, and Pelaez 1984) |
|  |  |  | *T. brasiliensis* | Yes | BRA - AL, BA, CE, PB, PI, RN, SE | P / D | Rocks, nests of rodents and houses. / Important vector in northeastern Brazil | (Neiva 1911a) |
|  |  |  | *T. breyeri* | No | ARG | P / OD | Rodents nests and environment Domiciliary | (Lent and Wygodzinsky 1979) |
|  |  |  | *T. carcavalloi* | Yes | BRA – RS | P / OD | Rocks and Occasionally found in houses | (Jurberg et al. 1998) |
|  |  |  | *T. carrioni* | Yes | ECU and PER | D | Domiciliary environment | (Larrousse 1926) |
|  |  |  | *T. cavernicola* | Yes | MYS | S | Caves | (Else et al. 1977) |
|  |  |  | *T. circummaculata* | Yes | URY and BRA – RS | P | Rocks and Cracks in stone walls | (Stål 1859) |
|  |  |  | *T. costalimai* | Yes | BOL and BRA - BA, GO, MG, MT, TO | P / OD | Rocks and invades houses but rarely colonizes | (Verano and Galvão 1958) |
|  |  |  | *T. deaneorum* | Yes | BRA – GO, MS | D | Domiciliary enviroment | (Galvão, Souza, and Lima 1967) |
|  |  |  | *T. delpontei* | No | BRA – RS | S | Nests of birds | (Romaña and Abalos 1947) |
|  |  |  | *T. dimidiata* | Yes | BLZ, COL, CRI, ECU, SLV, GUY, HND, MEX, NIC, PER and VEN | S / D | Various wild and domestic environments / Main vector of Central America. | (Latreille 1811) |
|  |  |  | *T. dispar* | Yes | ARG, BOL, PRY, URY and BRA- RS | S | Trees that have the presence of monkeys | (Lent 1950) |
|  |  |  | *T. eratyrusiformis* | Yes | ARG | P/ D | Rocks and occasionally in houses. | (Lent and Wygodzinsky 1979) |
|  |  |  | *T. garciabesi* | Yes | ARG | D | Domiciliary Environment | (Carcavallo et al. 1967) |
|  |  |  | *T. gerstaeckeri* | No | USA and MEX | S | Nests of mammals and occasionally domiciliary | (Stål 1859) |
|  |  |  | *T. gomeznunezi* | Yes | MEX | U | U | (Martinez, Carcavallo, and Jurberg 1994) |
|  |  |  | *T. guasayana* | Yes | ARG, BOL and PRY | P | Peridomiciliary environment | (Wygodzinsky and Abalos 1949) |
|  |  |  | *T. guazu* | Yes | PRY and BRA – MT | D | Domiciliary environment | (Lent and Wygodzinsky 1979) |
|  |  |  | *T. hegnri* | Yes | MEX | U | U | (Lent and Wygodzinsky 1979) |
|  |  |  | *T. incrassata* | Yes | USA and MEX | U | U | (Usinger 1939) |
|  |  |  | *T. indictiva* | Yes | USA and MEX | S | Wild environment | (Neiva 1912) |
|  |  |  | *T. infestans* | Yes | ARG, BOL, CHL, PRY, PER, URY and BRA- BA, RS | D/ P | Rocks, hollow trees, nests of birds and domiciliary enviroment. | (Klug 1834) |
|  |  |  | *T. jatai* | Yes | BRA – TO | P | Rocks and peridomiciliary environment. | (Gonçalves et al. 2013) |
|  |  |  | *T. juazeirensis* | Yes | BRA – BA, PI | P/OD | Rocks, peridocmiciliary and occasionally in houses. | (Costa and Felix 2007) |
|  |  |  | *T. jurbergi* | Yes | BRA – MT | P | Peridomiciliary environment | (Carcavallo, Galvão, and Lent 1998) |
|  |  |  | *T. klugi* | No | BRA – RS | S | Rock outcrops | (Carcavallo et al. 2001) |
|  |  |  | *T. lecticularia* | Yes | USA and MEX | D | Domiciliary Environment | (Stål 1859) |
|  |  |  | *T. lenti* | Yes | BRA – BA, GO | D/ P | Domiciliary environment and between rocks | (Sherlock and Serafim 1967) |
|  |  |  | *T. leopoldi* | Yes | AUS, IDN and PNG | U | U | (Schouteden 1933) |
|  |  |  | *T. limai* | Yes | ARG | S | Rocks | (Lent and Wygodzinsky 1979) |
|  |  |  | *T. longipennis* | Yes | MEX | D | Domiciliary Environment | (Usinger 1939)* |
|  |  |  | *T. maculata* | No | ABW, COL, GUY, SUR, VEN, BES, CUW and BRA – RR | P / D | Hollow trees, under bark of trees, nests, palms and domiciliary environment. | (Lent and Wygodzinsky 1979) |
|  |  |  | *T. matogrossensis* | Yes | BRA – MS | U | U | (Leite and Barbosa 1953) |
|  |  |  | *T. mazzottii* | No | MEX | D | Domiciliary Environment | (Usinger 1941) |
|  |  |  | *T. melanica* | Yes | BRA – MG | S | Rarely in houses | (Neiva and Lent 1941) |
|  |  |  | *T. melanocephala* | Yes | BRA - BA, PE, RN, SE | D | Domiciliary Environment | (Neiva and Pinto 1923a) |
|  |  |  | *T. mexicana* | Yes | MEX | D | Domiciliary Environment | (Lent and Wygodzinsky 1979) |
|  |  |  | *T. migrans* | Yes | IND, IDN, MYS, PHL, THA and Sarawak | S | Dead tree trunks | (Breddin 1903) |
|  |  |  | *T. mopan* | No | BLZ | W | Caves | (Dorn et al. 2018) |
|  |  |  | *T. neotomae* | No | USA | D | Domiciliary Environment | (Lent and Wygodzinsky 1979) |
|  |  |  | *T. nigromaculata* | Yes | COL and VEN | S | Trees, nests of birds and mammals | (Lent and Wygodzinsky 1979) |
|  |  |  | *T. nitida* | Yes | CRI, GUA, HND and MEX | D | Domiciliary Environment | (Usinger 1939) |
|  |  |  | *T. oliveirai* | No | BRA – RS | S | Nests of rodents - *Cavia aperea* | (Lent and Wygodzinsky 1979) |
|  |  |  | *T. pallidipennis* | Yes | MEX | D | Domiciliary Environment | (Lent and Wygodzinsky 1979) |
|  |  |  | *T. patagonica* | Yes | MEX | U | U | (Lent and Wygodzinsky 1979) |
|  |  |  | *T. peninsularis* | No | MEX | U | U | (Usinger 1940) |
|  |  |  | *T. petrocchiae* | Yes | BRA - BA, CE, PB, PE, RN | P / OD | Nests of rodents - *Kerodon rupestris* and ocasionally in houses. | (Pinto and Barreto 1925) |
|  |  |  | *T. phyllosoma* | Yes | MEX | D | Domiciliary Environment | (Lent and Wygodzinsky 1979) |
|  |  |  | *T. picturata* | Yes | MEX | U | U | (Usinger 1939) |
|  |  |  | *T. pintodiasi* | Yes | BRA – RS | P / S | Rocks and cracks in stone walls | (Jurberg et al. 2013) |
|  |  |  | *T. platensis* | Yes | ARG, BOL, PRY, URY and BRA – RS | S | Nests of birds (Passeriformes: Furnariidae) and occasionally in houses | (Neiva 1913) |
|  |  |  | *T. protracta* | Yes | USA and MEX | U | U | (Lent and Wygodzinsky 1979) |
|  |  |  | *T. pseudomaculata* | Yes | BRA - AL, BA, DF, CE, GO, MA, MS, PB, PE, PI, RN, SE, TO | P / D | Domiciliary and peridomiciliary associated with cacti and fences constructed with dry branches | (Corrêa and Spínola 1964) |
|  |  |  | *T. pugasi* | Yes | IDN | U | U | (Lent 1953a) |
|  |  |  | *T. recurva* | Yes | USA and MEX | U | U | (Lent and Wygodzinsky 1979) |
|  |  |  | *T. rubida* | No | MEX | U | U | (Lent and Wygodzinsky 1979) |
|  |  |  | *T. rubrofasciata* | Yes | ATG, CHN, GUF, HTI, HKG, IND, IDN, JAM, JPN, MYS, MRT, MMR, PNG, PHL, SAU, SLE,SGP, ZAF, LKA,VCT,TZA,THA, VEN,VNM,USA, AGO, ARG, BHS, KHM, CUB, DOM, COM, GRD, GLP, MTQ, REU, SYC, VCT, TWN, The Azores, Rodriguez Islands, Andaman Islands, Virgin Islands and BRA - CE, AL, BA, MA, PA, PB, PE, RJ, RN, SP, SE | S / P | Associated with synanthropic rodents | (Lent and Wygodzinsky 1979) |
|  |  |  | *T. rubrovaria* | No | ARG, URY and BRA – RS | P/ D | Rocks and Domiciliary Environment | (Lent and Wygodzinsky 1979) |
|  |  |  | *T. ryckmani* | No | GUA, HND and NIC | U | U | (Ryckman 1962) |
|  |  |  | *T. sanguisuga* | Yes | USA | D | Domiciliary Environment | (Lent and Wygodzinsky 1979) |
|  |  |  | *T. sherlocki* | Yes | BRA – BA | S | Rocks | (Pappa et al. 2002) |
|  |  |  | *T. sinaloensis* | Yes | USA and MEX | U | U | (Ramsey et al. 2015) |
|  |  |  | *T. sinica* | Yes | CHN | U | U | (Lent and Wygodzinsky 1979) |
|  |  |  | *T. sordida* | Yes | ARG, BOL, URY, PRY and BRA - BA, GO, MA, MT, MS, MG, PA, PE, PE, PI, PR, RS, SC, SE, SP, TO | S / P | Hollow trees, stacks of firewood, palms, henhouse, doves and currals. | (Stål 1859) |
|  |  |  | *T. tibiamaculata* | No | BRA - AL, BA, ES, MG, PE, RJ, SC, SP, SE | S | Nests of rodents, marsupials and palms | (Pinto 1926) |
|  |  |  | *T. vandae* | Yes | BRA – MT, MS | P | Peridomiciliary | (Carcavallo et al. 2002) |
|  |  |  | *T. venosa* | Yes | COL, ECU and PAN | U | U | (Lent and Wygodzinsky 1979) |
|  |  |  | *T. vitticeps* | Yes | BRA - BA, ES, MG, RJ | S / P | Nests of rodents, marsupials, henhouse and currals | (Stål 1859) |
|  |  |  | *T. williami* | Yes | BRA - GO, MS, MT | D | Domiciliary Environment | (Galvão, da Silva, and de Lima 1965) |
|  |  |  | *T. wygodzinskyi* | No | BRA – MG, SP | S | Rocks | (Lent 1951a) |

TS = total number of species in each genus; BS = number of species in each genus occurring in Brazil; H = habitat; D = domestic; OD = occasionally domestic; P = peridomiciliar; S = sylvatic; U = unknown.

# References

Abad-Franch, Fernando, Marcio G Pavan, Nicolas Jaramillo-O, Francisco S Palomeque, Carolina Dale, Duverney Chaverra, and Fernando A Monteiro. 2013. "Rhodnius barretti, a new species of Triatominae (Hemiptera: Reduviidae) from western Amazonia." *Memórias do Instituto Oswaldo Cruz* 108:92-99.

Aguilar, Ricardo Alejandre, Benjamín Nogueda Torres, Máximo Cortéz Jímenez, José Jurberg, Cleber Galvão, and Rodolfo Carcavallo. 1999. "Triatoma bassolsae sp. n. from Mexico with a key to species of" phyllosoma" complex." *Memórias do Instituto Oswaldo Cruz* 94 (3):353-359.

Almeida, Flávio Barbosa de, Elcy Israel Santos, and Gigio Sposina. 1973. "Triatomíneos da Amazônia III.()." *Acta Amazônica* 3 (2):43-46.

Ayala, José Manuel. 2009. "Una nueva especie de Panstrongylus Berg de Venezuela (Hemiptera: Reduviidae, Triatominae)." *Entomotropica* 24 (3):105-109.

Barber, HG. 1932. "A new species of Rhodnius from Panama (Hemiptera: Reduviidae)." *Journal of the Washington Academy of Sciences* 22 (18/19):514-517.

Barber, HG. 1937. "A new bat-cave bug from Panama (Hemiptera, Heteroptera: Reduviidae)." *Proceedings of the Entomological Society of Washington* 39:61-63.

Barber, HG. 1938. "A new genus and species of the subfamily Triatominae (Reduviidae: Hemiptera)." *Proceedings of the Entomological Society of Washington* 40:104-105.

Barrett, Toby V, and Jorge R Arias. 1985. "A new triatomine host of Trypanosoma from the Central Amazon of Brazil: Cavernicola lenti n. sp.(Hemiptera, Reduviidae, Triatominae)." *Memórias do Instituto Oswaldo Cruz* 80 (1):91-96.

Berg, C. 1879. "Hemiptera Argentina enumeravit species que novas descripsit." *PE Coni, Buenos Aires, Argentina*.

Breddin, G. 1903. "Neue Paläotropische Reduviinen." *Gesellschaft Naturforschender Freunde, Berlin* 3:111-129.

Bérenger, Jean-Michel, and Denis Blanchet. 2007. "A new species of the genus Panstrongylus from French Guiana (Heteroptera; Reduviidae; Triatominae)." *Memórias do Instituto Oswaldo Cruz* 102 (6):733-736.

Carcavallo, R, and P Barreto. 1976. "A new species of Rhodnius in Colombia." *Boletín de la Dirección de Malariologia y Saneamiento Ambiental* 16 (2):176-183.

Carcavallo, R. U., J. Jurberg, D.a S Rocha, C. Galvao, F. Noireau, and H. Lent. 2002. "[Triatoma vandae sp.n. of the oliveirai complex from the State of Mato Grosso, Brazil (Hemiptera: Reduviidae: Triatominae)]." *Mem Inst Oswaldo Cruz* 97 (5):649-54.

Carcavallo, Rodolfo U, Cleber Galvão, and Herman Lent. 1998. "Triatoma jurbergi sp. n. do norte do estado do Mato Grosso, Brasil (Hemiptera, Reduviidae, Triatominae) com uma atualização das sinonímias e outros táxons." *Memórias do Instituto Oswaldo Cruz* 93 (4):459-464.

Carcavallo, Rodolfo U, José Jurberg, Herman Lent, Cleber Galvão, Mário Steindel, and Carlos José Carvalho Pinto. 2001. "Nova espécie do complexo oliveirai (nova denominação para o complexo matogrossensis)(Hemiptera, Reduviidae, Triatominae) do Estado do Rio Grande do Sul, Brasil." *Mem Inst Oswaldo Cruz* 96 (1):71-9.

Carcavallo, RU, JA Cichero, A Martínez, AF Prosen, and R Ronderos. 1967. "Una nueva especie del género Triatoma Laporte (Hemiptera, Reduviidae, Triatominae)." *Segundas J Entomol Arg* 2:43-8.

Carcavallo, RU, and J Jurberg. 2000. "Triatoma baratai sp. n. from the state of Mato Grosso do Sul, Brazil (Hemiptera, Reduviidae, Triatominae)." *Entomologia y Vectores* 7 (4):373-387.

Corrêa, RR, and HN Spínola. 1964. "Description of Triatoma pseudomaculata, a new species of Triatominae of Sobral, Ceará (Hemiptera, Reduviidae)." *Arquivos de higiene e saude publica* 29 (101):115.

Costa, Jane, and Márcio Felix. 2007. "Triatoma juazeirensis sp. nov. from the state of Bahia, Northeastern Brazil (Hemiptera: Reduviidae: Triatominae)." *Memórias do Instituto Oswaldo Cruz* 102 (1):87-90.

da Rosa, J. A., H. H. G. Justino, J. D. Nascimento, V. J. Mendonça, C. S. Rocha, D. B. de Carvalho, R. Falcone, M. T. V.A Oliveira, K. C. C. Alevi, and J. de Oliveira. 2017. "A new species of Rhodnius from Brazil (Hemiptera, Reduviidae, Triatominae)." *Zookeys* (675):1-25. doi: 10.3897/zookeys.675.12024.

Distant, WL. 1903. 1904: The Fauna of British India, Including Ceylon and Burma. Rhyn. 2. Taylor and Francis, London.

Dorn, P. L., Justi, S. A., Dale, C., Stevens, L., Galvão, C., Lima-Cordón, R., and Monroy, C. 2018. "Description of Triatoma mopan sp. n. from a cave in Belize (Hemiptera, Reduviidae, Triatominae)." *ZooKeys* (775): 69.

Else, JG, WH Cheong, S Mahadevan, and LG Zarate. 1977. "A new species of cave-inhabiting Triatoma (Hemiptera: Reduviidae) from Malaysia." *Journal of Medical Entomology* 14 (3):367-369.

Fernández-Loayza, Roberto. 1989. "Triatoma matsunoi nueva especie del norte peruano (Hemiptera, Reduviidae: Triatomidae)." *(Dic 1988) v. 31 p. 21-24*.

Frias, Daniel A, ABEL A HENRY, and Christian R Gonzalez. 1998. "Mepraia gajardoi: a new species of Triatominae (Hemiptera: Reduviidae) from Chile and its comparison." *Revista Chilena de Historia Natural* 71:177-188.

Frías-Lasserre, Daniel. 2010. "A new species and karyotype variation in the bordering distribution of Mepraia spinolai (Porter) and Mepraia gajardoi Frías et al (Hemiptera: Reduviidae: Triatominae) in Chile and its parapatric model of speciation." *Neotropical entomology* 39 (4):572-583.

Galvao, Cleber, and Victor M Angulo. 2006. "Belminus corredori, a new species of Bolboderini (Hemiptera: Reduviidae: Triatominae) from Santander, Colombia." *Zootaxa* 1241 (1):61-68.

Galvão, AB, E SOUZA DA SILVA, and RR DE LIMA. 1965. "Triatoma williami n. sp.(Hemiptera, Triatominae)." *Revista Brasileira de Malariologia* 17 (4):363-6.

Galvão, AB, and JD Palma. 1968. "Uma nova espécie do genero Panstrongylus berg, 1879 (Reduviidae, Triatominae)." *Revista Brasileira da Biologia* 28:403-405.

Galvão, AB, HA da S Souza, and RR de Lima. 1967. "Espécies de Triatominae ocorrentes em Goiás e descrição de uma nova espécie." *Revista Brasileira de Malariologia e Doenças Tropicais* 19:397-412.

Galvão, C, JS Patterson, D Da Silva Rocha, J Jurberg, R Carcavallo, K Rajan, DP Ambrose, and MA Miles. 2002. "A new species of Triatominae from Tamil Nadu, India." *Medical and veterinary entomology* 16 (1):75-82.

Ghauri, MSK. 1976. "The Indian triatomine genus Linshcosteus (Reduviidae)." *Systematic Entomology* 1 (3):183-187.

Gonçalves, Teresa Cristina Monte, Simone Caldas Teves-Neves, Jacenir Reis dos Santos-Mallet, Ana Laura Carbajal-de-la-Fuente, and Catarina Macedo Lopes. 2013. "Triatoma jatai sp. nov. in the state of Tocantins, Brazil (Hemiptera: Reduviidae: Triatominae)." *Memórias do Instituto Oswaldo Cruz* 108 (4):429-437.

Herrer, A, H Lent, and Pedro Wygodzinsky. 1954. "Contribución al conocimiento del género BelminusStal, 1859 (Triatominae, Reduviidae, Hemiptera)." *An Inst Med Reg Univ Tucuman* 4:85-106.

Jurberg, J, DS Rocha, ES Lorosa, MC Vinhaes, and H Lent. 1998. "Uma nova espécie de Triatoma do estado do Rio Grande do Sul, Brasil (Hemiptera, Reduviidae)."

Jurberg, José, Vanda Cunha, Solange Cailleaux, Raquel Raigorodschi, Michele Souza Lima, Dayse da Silva Rocha, and Felipe Ferraz Figueiredo Moreira. 2013. "Triatoma pintodiasi sp. nov. do subcomplexo T. rubrovaria (Hemiptera, Reduviidae, Triatominae)." *Revista Pan-Amazônica de Saúde* 4 (1):43-56.

Jurberg, José, Dayse da Silva Rocha, and Cleber Galvão. 2009. "Rhodnius zeledoni sp. nov. afim de Rhodnius paraensis Sherlock, Guitton & Miles, 1977 (Hemiptera, Reduviidae, Triatominae)." *Biota Neotropica* 9 (1):0-0.

Klug, F. 1834. "In Reise um die Erde. In den Jahren 1830, 1831, und 1832 ausgefuert von FJF Meyen." *Teil* 1:412.

Larrousse, F. 1924. "Triatomes d’Asie; description d’une nouvelle espèce Triatoma bouvieri n. sp." *Annales de Parasitologie Humaine et Comparée* 2 (1):62-70.

Larrousse, F. 1926. "Description de deux espèces nouvelles du genre Triatoma: T. carrioni n. sp., et T. pintoi n. sp." *Annales de Parasitologie Humaine et Comparée* 4 (2):136-139.

Larrousse, F. 1927. Etude biologique et systématique du genre Rhodnius Stal (Hémiptères, Reduvidæ). EDP Sciences.

Latreille, Piérre André. 1811. "Insectes de l’Amérique recueillis pendant le voyage de MM. de Humboldt et Bonpland." *Voyage aux régions equinoxiales du nouveau continent* 1:197-397.

Leite, IC, and A Barbosa. 1953. "Triatoma (Eutriatoma) matogrossensis n. sp." *Boletim do Instituto Oswaldo Cruz* 2:1-3.

Lent, H. 1942. "Estudos sobre os triatomíneos do Estado do Rio Grande do Sul, com descrição de uma espécie nova." *Rev Bras Biol* 2:219-231.

LENT, H. 1943. "A New Transmitter of Chagas's Disease in the Town of Rio de Janeiro, Parabelminus carioca." *Memorias do Instituto Oswaldo Cruz* 38 (3):497-516.

Lent, H. 1950. "Nova espécie de Triatoma Laporte, 1833 (Hemiptera, Reduviidae)." *Rev Brasil Biol* 10:437-440.

Lent, H. 1951a. "Novo Triatoma no Estado de Minas Gerais (Brasil)(Hemiptera: Reduviidae)." *Revista de Entomologia* 22 (1-3):349-353.

Lent, H. 1953a. "Nova especie de Triatoma da regiao Oriental (Hemiptera, Reduviidae)." *Rev. Bras. Biol* 13 (3):5.3.

Lent, H. 1953b. "Um novo hemiptero hematófago da Venezuela (Reduviidae, Triatominae)." *Rev Bras Biol* 13:169-72.

Lent, H, and J Jurberg. 1965. "O gênero Psammolestes Bergroth, 1911, com um estudo sôbre a genitália das espécies (Hemiptera, Reduviidae, Triatominae)." *Revista brasileira de Biología* 25:344-376.

Lent, H, and AV Martins. 1940. "Estudos sobre os triatomideos do Estado de Minas Gerais, com descrição de uma espécie nova." *Rev Entomol* 11:877-886.

Lent, H., and P. Wygodzinsky. 1979. "Revision of the Triatominae (Hemiptera, Reduviidae) and their significanse as vectors of Chagas disease.    ." *Bull. Am. Mus. Nat. Hist.* 163:125-520.

Lent, Herman. 1951b. "Triatominae das regioes Oriental, Australiana, Etiopica e Paleartica, com descricao de uma nova especie (Hemiptera, Reduviidae)." *Revista Brasileira de Biologia* 11:425-429.

Lent, Herman. 1954. "Comentários sobre o gênero Rhodnius Stal, com descrição de uma nova espécie do Brasil (Hemiptera, Reduviidae)." *Rev Bras Biol* 14:237-247.

Lent, Herman, José Jurberg, and Rodolfo Ubaldo Carcavallo. 1995. "Belminus laportei sp. n. from the Amazon Region (Hemiptera: Reduviidae: Triatominae)." *Memórias do Instituto Oswaldo Cruz* 90 (1):33-39.

Lent, Herman, José Jurberg, and Cleber Galvão. 1993. "Rhodnius stali n. sp. related to Rhodnius pictipes Stal, 1872 (Hemiptera, Reduviidae, Triatominae)." *Memórias do Instituto Oswaldo Cruz* 88 (4):605-614.

Lent, HERMAN, and LUIS A León. 1958. "Um novo Rhodnius Stäl do Ecuador (Hemiptera, Reduviidae)." *Rev Bras Biol* 18:181-185.

Maldonado, J, and TH Farr. 1962. "On some Jamaican Triatominae and Emesinae." *Proceedings of the Entomological Society of Washington* 64:187-194.

Martinez, Antonio, Rodolfo U Carcavallo, and Jose Jurberg. 1994. "Triatoma gomeznunezi a new species of Triatomini from Mexico (Hemiptera, Reduviidae, Triatominae)." *Entomología y Vectores* 1:15-19.

Martinez, E, T Chávez, D Sossa, R Aranda, B Vargas, and P Vidaurre. 2007. "Triatoma boliviana sp. n. de los valles subandinos de La Paz, Bolivia (Hemiptera: Reduviidae: Triatominae), similar a Triatoma nigromaculata Stål, 1859." *Bol Inst Invest Salud Desar* 3:1-11.

Martínez, A, RU Carcavallo, and D Pelaez. 1984. "Triatoma brailovskyi, nueva especie de triatominae de México." *Chagas* 1 (2):39-42.

Martínez, Antonio, and FU Carcavallo. 1977. "Un nuevo triatominae neotropical (Hemíptera: Reduviidae)." *Folia Entomologica Mexicana*.

Matta, A. 1919. "Um novo reduvídeo do Amazonas, Rhodnius brethesi n. sp." *Amazonas Med* 2:93-94.

Mejia, JM, C Galvão, and J Jurberg. 1999. "Rhodnius colombiensis sp. n. da Colombia com quadros comparativos entre as estruturas fálicas do gênero Rhodnius Stal 1859 (Hemiptera, Reduviidae, Triatominae)."

Neiva, A. 1911a. "Contribuição para o estudo dos hematophagos brazileiros e descrição de uma nova espécie de Triatoma." *Brasil Médico* 25:461-462.

Neiva, A. 1911b. "Notas de entomología médica. Tres novas especies de redúvidas norte-americanas." *Brasil-Médico* 25:441.

Neiva, A. 1912. "Notas de entomología médica e descripção de duas novas espécies de Triatomas norte-americanas." *Brasil-Médico* 26:21-22.

Neiva, A. 1913. "Algunos datos sobre hemípteros hematófagos de la América del sur, con la descripción de una nueva especie." Anales del Museo Nacional de Historia Natural, Buenos Aires.

NEIVA, A, and H LENT. 1941. "A catalogue of triatomids." *Revista de Entomologia* 12 (pt. 1-2):61-92.

Neiva, A, and C Pinto. 1923a. "Dos hemípteros hematophagos do Norte do Brasil com descrição de duas novas espécies." *Brasil Médicina* 37:73-76.

Neiva, A, and C Pinto. 1923b. "Estado actual dos conhecimentos sobre o gênero Rhodnius Stål, com a descrição de uma nova espécie." *Bras Med* 37:20-24.

Osuna, Eduardo, and José Manuel Ayala. 1993. "Belminus pittieri, nueva especie de Bolboderini (Triatominae: Reduviidae: Heteroptera)." *Boletín Entomología Venezolana* 8:147-150.

PAPPA, AR, J Jurberg, RU Carcavallo, RL Cerqueira, and JMS Barata. 2002. "Triatoma sherlocki sp. n. coletada na Bahia, Brasil (Hemiptera, Reduviidae, Triatominae)."

Pinto, C. 1926. "Triatomideos da Venezuela, com a descripção de uma nova espécie do gênero Eutriatoma." *Ann. Fac. Med. São Paulo* 1:85-87.

Pinto, C, and JB Barreto. 1925. "Uma nova espécie de “barbeiro” do Brasil,(Triatoma petrochii n. sp.)." *Sciencia Medica* 3:769.

Pinto, C, and H Lent. 1946. "Novo hemíptero hematófago do gênero “Panstrongylus” Berg, 1879." *Rev. Bras. Biol* 6:459-465.

Porter, CE. 1934. "Una Triatoma nueva chilena." *Revista Chilena de Historia Natural* 37:192-193.

Ramsey, J. M., A. T. Peterson, O. Carmona-Castro, D. A. Moo-Llanes, Y. Nakazawa, M. Butrick, E. Tun-Ku, K. la Cruz-Félix, and C. N. Ibarra-Cerdeña. 2015. "Atlas of Mexican Triatominae (Reduviidae: Hemiptera) and vector transmission of Chagas disease." *Mem Inst Oswaldo Cruz* 110 (3):339-52. doi: 10.1590/0074-02760140404.

Romaña, C, and J Abalos. 1947. "Triatoma delpontei n. sp.(Hemiptera, Reduviidae)." *An. Inst. Med. Regional* 2 (1).

Rosa, JA da, Claudia Solano Rocha, Sueli Gardim, Mara Cristina Pinto, Vagner Jose Mendonca, JCR Ferreira Filho, EOC Carvalho, LM Aranha Camargo, J Oliveira, and Juliana Damieli Nascimento. 2012. "Description of Rhodnius montenegrensis n. sp.(Hemiptera: Reduviidae: Triatominae) from the state of Rondônia, Brazil." *Zootaxa* 3478:62-76.

Ryckman, Raymond E. 1962. *Biosystematics and hosts of the Triatoma protracta complex in North America,(Hemiptera: Reduviidae; Rodentia: Cricetidae)*: University of California Press.

Sandoval, Claudia Magaly, Eulides Pabon, Jose Jurberg, and Cleber Galvao. 2007. "Belminus ferroae n. sp. from the Colombian north-east, with a key to the species of the genus (Hemiptera: Reduviidae: Triatominae)." *Zootaxa* 1443 (1):55-64.

Schouteden, H. 1933. "Hemiptera-Heteroptera." *Résultats Scientifiques du Voyage aux Indes Orientales Néerlandaises de LL. AA. RR. le Prince et la Princesse Léopold de Belgique. Hemiptera-Heteroptera. Mémoires du Musée Royal d’Histoire Naturelle de Belgique, Hors Série* 4 (8):1-70.

Serra, RG, NCB Atzingen, and OP Serra. 1987. "Nueva especie del género Alberprosenia Martínez & Carcavallo, 1977, del Estado de Pará, Brasil." *Chagas* 4 (1):3.

Sherlock, IA, and EM Serafim. 1967. "Triatoma lenti sp. n." *Triatoma pessoai*:75-92.

Sherlock, Ítalo A, Neide Guitton, and Michael Miles. 1977. "Rhodnius paraensis espécie nova do Estado do Pará, Brasil (Hemiptera, Reduviidae, Triatominae)." *Acta Amazônica* 7 (1):71-74.

Souza, ED, NC Von Atzingen, Maria Betânia Furtado, Jader de Oliveira, Juliana Damieli Nascimento, Daniel Pagotto Vendrami, Sueli Gardim, and João Aristeu da Rosa. 2016. "Description of Rhodnius marabaensis sp. n.(Hemiptera, Reduviidae, Triatominae) from Pará State, Brazil." *ZooKeys* (621):45-62.

Stål, C. 1859. "Monographie der gattung conorhinus und verwandten." *Berliner Entomologische Zeitschrift* 3 (2‐3):99-117.

Uhler, Philip Reese. 1894. "Observations upon the heteropterous hemiptera of Lower California."

Usinger, R. 1941. "Notes and descriptions of neotropical Triatominae (Hemiptera, Reduviidae)." *Pan-Pacific Entomol* 17 (2):49-57.

Usinger, RL. 1940. "A new Triatoma from Lower California (Hemiptera, Reduviidae)." *Pan-Pacific Entomologist* 16:73-74.

Usinger, Robert Leslie. 1939. "Descriptions of new Triatominae with a Key to Genera (Hemiptera, Reduviidae)." *Univiversity of California Publications in Entomology* 7 (3).

Usinger, Robert Leslie. 1944. "The Triatominae of North and Central America and the West Indies and their public health significance." *The Triatominae of North and Central America and the West Indies and their Public Health Significance.* (288).

Valdés, P. 1910. "Clasificación Gundlach de Hemipteros Cubanos, conforme a los ejemplares que existen en el Museo del Instituto de 2a enseñanza de La Habana." Anales de la Academia de Ciencias Médicas, Físicas y Naturales de la Habana.

Valente, Vera da Costa, Sebastião Aldo da Silva Valente, Rodolfo Ubaldo Carcavallo, Dayse da Silva Rocha, Cleber Galvão, and José Jurberg. 2001. "Considerações sobre uma nova espécie do gênero Rhodnius stal, do Estado do Pará, Brasil (Hemiptera, Reduviidae, Triatominae." *Entomol. vectores* 8 (1):65-80.

Verano, OT, and Archibaldo Bello Galvão. 1958. "Triatoma costalimai sp., n." *Rev. bras. Malar* 10:199-205.

Wygodzinsky, P, and JW Abalos. 1949. "Triatoma guasayana sp. n.(Triatominae, Reduviidae, Hemiptera)(Nota previa)." *Semana Médica, Buenos Aires* 56 (2).
